# Anticipated stigma and perceived social support among maintenance hemodialysis patients in Syria: a multicentre cross-sectional study in a conflict-affected setting

**DOI:** 10.1186/s40359-026-04710-3

**Published:** 2026-05-11

**Authors:** Omar Al Ayoubi, Mohammad Alaa Aldakak, Grace Tannous, Alaa Senjab, Ghena Arar, Baraah Bani Almarjeh, Maria Kassoumeh, Omar Hafez, Youssef Latifeh

**Affiliations:** 1 Faculty of Medicine, Damascus University, Damascus, Syrian Arab Republic; 2 Faculty of Medicine, Latakia University, Latakia, Syrian Arab Republic; 3 Department of Psychiatry, Faculty of Medicine, Damascus University, Damascus, Syrian Arab Republic

**Keywords:** Anticipated stigma, Perceived social support, Hemodialysis, End-stage kidney disease, Chronic illness, Conflict-affected settings, Cross-sectional study

## Abstract

**Importance:**

Stigma is a significant psychosocial challenge among patients receiving maintenance hemodialysis (HD). Evidence from conflict-affected, resource-limited settings is scarce, particularly regarding anticipated stigma, perceived social support, and site-level differences in dialysis care.

**Objective:**

To assess the prevalence of anticipated stigma and its association with perceived social support and clinical factors among maintenance HD patients in Syria.

**Design, setting, and participants:**

This multicenter cross-sectional study was conducted from June 30 to December 4, 2025, among 507 adult patients receiving maintenance hemodialysis across five Syrian governorates. Data were collected using interviewer-administered questionnaires.

**Exposures:**

Perceived social support measured by the Multidimensional Scale of Perceived Social Support (MSPSS), and clinical and dialysis-related characteristics, including blood transfusion history, erythropoietin therapy, chronic pruritus, perceived dialysis-related financial burden, and hemodialysis hospital/governorate.

**Main outcomes and measures:**

The primary outcome was anticipated stigma, defined operationally as a Chronic Illness Anticipated Stigma Scale (CIASS) total score > 24. Secondary measures included CIASS and Multidimensional Scale of Perceived Social Support (MSPSS) total and domain scores. Analyses included Spearman correlations, nonparametric group comparisons, hierarchical block-wise binary logistic regression, and a sensitivity analysis treating CIASS total score as a continuous variable.

**Results:**

The mean age was 48.3 years (SD 14.9), and 57.0% of participants were male. Anticipated stigma was identified in 39.6% of patients, with a median CIASS score of 24 (IQR 19–28). Median MSPSS score was 67 (IQR 58–73), and 66.9% of participants reported high perceived social support. CIASS and MSPSS scores were inversely correlated (rₛ = −0.170, *p* < 0.001). In the fully adjusted hierarchical model, blood transfusion history was independently associated with higher odds of anticipated stigma (OR 2.09, 95% CI 1.27–3.45), while higher MSPSS score was associated with lower odds (OR 0.965, 95% CI 0.949–0.983). Hemodialysis hospital/governorate remained significantly associated with anticipated stigma in the final model (*p* < 0.001). Sensitivity analysis using CIASS as a continuous outcome showed consistent direction of associations.

**Conclusions and relevance:**

Anticipated stigma was common among maintenance HD patients in this conflict-affected setting. Higher perceived social support was consistently associated with lower anticipated stigma, whereas blood transfusion history was associated with higher stigma. These findings should be interpreted as associative and exploratory rather than predictive, and do not represent a clinical screening or prediction tool. Future research should address psychological and institutional determinants to better explain stigma mechanisms in this population.

## Introduction

Chronic kidney disease (CKD) is one of the fastest-growing non-communicable diseases (NCDs) worldwide and is defined as a persistent abnormality in kidney structure or function lasting more than three months [1, 2]. Its increasing prevalence poses a substantial socioeconomic and public health burden, particularly in resource-limited settings [3]. According to the Global Burden of Disease (GBD) study, CKD ranked as the 11th leading cause of death in 2019, rising from 19th in 1990, accounting for 2.53% of all deaths [4]. With population aging and the growing prevalence of hypertension and diabetes, the number of patients receiving maintenance hemodialysis (HD) has increased markedly [5]. Although advances in dialysis have improved survival among patients enrolled in long-term programs [5], HD does not fully replace normal renal function, and patients frequently experience physical complications such as pruritus, bone pain, skin pigmentation, restless leg syndrome, and sleep disturbances alongside psychological distress, including anxiety, depression, and stigma, affecting quality of life [6]. Notably, approximately 45% of adults undergoing HD report varying degrees of psychological problems [7]. These challenges are further intensified in conflict-affected and resource-limited settings, where damaged healthcare infrastructure and limited access to essential services disproportionately affect patients with end-stage kidney disease (ESKD) [8]. In Syria, for example, the reported prevalence of ESKD varies widely, ranging from 55 to 818 cases per million population [9]. Patients in such settings face additional psychosocial stressors, including economic instability, disrupted healthcare services [9]. Among the psychosocial burdens faced by hemodialysis patients, stigma is particularly pervasive. Physical appearance changes related to CKD and long-term dialysis such as skin hyperpigmentation, uremic frost, desquamation, fistulas, and ammoniacal breath odor combined with diminished social status, reduced employment opportunities, and public misconceptions about uremia, often contribute to stigmatization [10, 11]. Stigma involves self-stigmatization and internalization of social discrimination, undermining self-perception, restricting social interactions, and worsening psychological distress [10]. It can manifest as enacted, perceived, or anticipated stigma; anticipated stigma refers to expectations of prejudice or discrimination in the future, while perceived stigma reflects awareness of negative societal stereotypes both are prevalent and severe among HD patients [11, 12]. In this context, social support including emotional, informational and practical assistance from family, friends and healthcare providers mitigates the psychological impact of stigma among HD patients [13–15].

Despite growing recognition of stigma and social support as key psychosocial determinants of health, empirical evidence examining their relationship in conflict-affected and resource-limited settings remains limited. This gap is particularly evident in Syria, where prolonged conflict, disrupted healthcare infrastructure, and complex social conditions may intensify psychosocial burdens among patients with end-stage kidney disease (ESKD). Accordingly, this cross-sectional study aimed to evaluate anticipated stigma and perceived social support, examine their association, and explore related sociodemographic and clinical factors among patients receiving maintenance hemodialysis in Syria.

## Methods

### Study design and setting

This multicenter cross-sectional study was conducted between June 30 and December 4, 2025, among maintenance hemodialysis patients recruited from hemodialysis units across five Syrian governorates: Damascus and its suburbs, Aleppo, Homs, Hama, and Latakia. The study protocol was approved by the Institutional Review Board of Damascus University (Approval No. MD-300625-474), and all participants provided written informed consent before enrollment.

### Participant eligibility criteria and sampling

Consecutive adult patients aged 18 years or older who were receiving maintenance hemodialysis at participating dialysis units during the study period were invited to participate. Inclusion criteria were: a confirmed diagnosis of end-stage renal disease treated with maintenance hemodialysis, regular attendance at the dialysis unit, ability to communicate in Arabic, and capacity to provide informed consent.

Patients were excluded if they had acute medical instability at the time of data collection, such as hemodynamic instability or hospitalization; documented cognitive impairment, severe psychiatric illness, or neurological conditions interfering with reliable questionnaire completion; or refusal or inability to provide informed consent.

### Data collection procedure

Data were collected using a structured interviewer-administered questionnaire during face-to-face interviews conducted by trained research interviewers, including medical students and nurses. Interviews were performed during dialysis sessions or immediately before or after the session.

The questionnaire comprised three sections. The first section captured sociodemographic characteristics, including sex, age, marital status, educational attainment, smoking status, employment status, self-reported economic status, governorate of residence, displacement status, household size, and number of children. The second section addressed hemodialysis-related and clinical characteristics, including hemodialysis hospital/governorate, dialysis shift, presence of a companion during dialysis, dialysis vintage in months, duration of a single dialysis session in hours, number of sessions per month, travel time to the dialysis center in minutes, perceived financial burden of dialysis and its severity rating, comorbidities such as hypertension and diabetes, chronic pruritus and its severity, hemoglobin level, history of blood transfusion, history and frequency of dialysis- or hospital-related infections, regular physician follow-up, renal diet awareness and adherence, and current erythropoietin therapy. The third section included the standardized psychosocial instruments: the Multidimensional Scale of Perceived Social Support (MSPSS) and the Chronic Illness Anticipated Stigma Scale (CIASS).

### Measures

#### Perceived social support (MSPSS)

 Perceived social support was assessed using the Multidimensional Scale of Perceived Social Support (MSPSS), a 12-item instrument evaluating perceived support from three sources: family, friends, and a significant other. Items are rated on a 7-point Likert scale, from 1 = very strongly disagree to 7 = very strongly agree. The total MSPSS score was computed as the sum of all 12 items, ranging from 12 to 84, with higher scores indicating greater perceived social support. For descriptive analyses, MSPSS total scores were categorized as low support (≤ 35), medium support (36–60), and high support (61–84), using commonly applied cut points [16].

#### Anticipated stigma (CIASS) 

Anticipated stigma related to chronic illness was measured using the 12-item Chronic Illness Anticipated Stigma Scale (CIASS), which evaluates anticipated stigma from three domains: friends and family, work colleagues, and healthcare workers. Items are rated on a 5-point Likert scale, from 1 = very unlikely to 5 = very likely. The CIASS total score was computed as the sum of all items, ranging from 12 to 60, with higher scores indicating greater anticipated stigma [12].

For categorical analyses, participants were classified as having anticipated stigma if their CIASS total score was > 24, and as having no anticipated stigma if their score was ≤ 24. This threshold was used as an operational cut point consistent with prior CIASS-based work [17]; however, because the CIASS was originally developed and psychometrically evaluated primarily as a continuous scale, we also conducted a sensitivity analysis treating CIASS total score as a continuous outcome.

The full 12-item CIASS was administered to all participants to preserve the original scale structure and scoring. No employment-status-specific skip logic was applied. Therefore, the work colleagues domain was retained for all participants, including those who were retired or had never worked, and current employment status was accounted for in the multivariable model.

### Translation and linguistic procedures

Both instruments were originally developed in English and were translated into Arabic by a professional translator, then independently back-translated into English. Discrepancies were reviewed and resolved by the study team to support semantic and conceptual equivalence. Cognitive debriefing and pilot testing were conducted on a convenience sample of 50 hemodialysis patients to assess clarity, comprehension, and completion time. Minor wording adjustments were made before full data collection.

### Reliability

Internal consistency was assessed using Cronbach’s alpha and standardized alpha. In the present sample of 507 complete cases, the CIASS demonstrated good internal consistency, with α = 0.850 and standardized α = 0.856 across 12 items. The MSPSS also demonstrated good internal consistency, with α = 0.878 and standardized α = 0.894 across 12 items. The present study assessed internal consistency but did not conduct a full construct validation of the Arabic versions of the CIASS or MSPSS in this hemodialysis population.

### Statistical Analysis

Statistical analyses were performed using IBM SPSS Statistics, version 26.0 (IBM Corp., Armonk, NY, USA). Categorical variables were summarized as frequencies and percentages. Continuous variables were summarized as mean and standard deviation for approximately symmetric distributions, or median and interquartile range for skewed distributions. Scale totals and subscale scores were computed according to published scoring rules, and categorical classifications were derived where applicable, including MSPSS support categories and CIASS anticipated-stigma status.

Bivariate associations between continuous scale scores and continuous demographic or clinical variables were examined using Spearman’s rank correlation coefficient. Group comparisons for two-category predictors were performed using the Mann–Whitney U test, with effect size reported as r derived from the standardized test statistic. Comparisons across three or more categories were performed using the Kruskal–Wallis test, with mean ranks reported. For ordinal predictors, trend across ordered groups was additionally evaluated using the Jonckheere–Terpstra test, with the standardized Z statistic and effect size r reported. All tests were two-sided, and statistical significance was set at *p* ≤ 0.05.

Multivariable analyses were performed using hierarchical block-wise binary logistic regression to identify independent correlates of anticipated stigma, defined as CIASS total score > 24. Predictor entry was guided by conceptual relevance and prior literature rather than by statistical significance in bivariate analyses alone. Variables were entered in four sequential blocks. Block 1 included sociodemographic variables: age, number of household members, educational attainment, economic status, employment-status indicators, and displacement status. Block 2 added clinical and dialysis-related variables: perceived financial burden of dialysis, chronic itching, erythropoietin therapy, and history of blood transfusion. Block 3 added hemodialysis hospital/governorate as a site-level covariate to account for potential differences across dialysis settings. Block 4 added perceived social support, represented by MSPSS total score.

Results are reported as odds ratios with 95% confidence intervals derived from the estimated regression coefficients. Categorical predictors were entered using indicator coding with appropriate reference categories. Incremental model contribution was evaluated using changes in − 2 log-likelihood, likelihood-ratio χ² tests, and pseudo-R² indices, including Cox & Snell R² and Nagelkerke R². Model calibration was assessed using the Hosmer–Lemeshow goodness-of-fit test, and discriminatory performance was summarized using the classification table, including overall accuracy, sensitivity, and specificity. Multicollinearity was examined using tolerance and variance inflation factors.

To address potential information loss from dichotomizing the CIASS total score, an additional sensitivity analysis was conducted using CIASS total score as a continuous outcome. This model included the same covariates as the final logistic regression model and used heteroskedasticity-consistent robust standard errors (HC3). This analysis assessed whether the main associations observed in the logistic regression model were consistent when the full variability of the CIASS score was retained.

## Results

### Sample characteristics and dialysis-related characteristics

A total of 507 hemodialysis patients were included, of whom 57.0% were male. The mean age was 48.28 ± 14.90 years. Most participants were married (77.9%), and the median household size was 4 (IQR 3–5). Educational attainment was broadly distributed; nearly half of the participants reported an average economic status (48.3%), while 39.1% reported below-average economic status. Participants were recruited across multiple governorates, and 15.0% reported forced displacement. Dialysis was commonly described as a financial burden (53.5%). The median hemodialysis duration was 36 months (IQR 18–72), with a typical schedule of 8 sessions per month (IQR 8–8) and a median travel time to the dialysis center of 30 min (IQR 20–45) (Table 1).

**Table 1:** Participant characteristics & Descriptive results for study scales (*N* = 507)

Characteristic	Category / statistic	Value	*N*
Sex, n (%)	Male	289 (57.0)	507
	Female	218 (43.0)	
Age (years)	**Mean (SD)**	**48.28 ± 14.90**	507
Marital status, n (%)	Currently married	395 (77.9)	507
	Currently unmarried	112 (22.1)	
Employment status, n (%)	Office worker/office job	110 (21.7)	507
	Retired	182 (35.9)	
	Never worked	117 (23.1)	
	A job that requires physical effort	98 (19.3)	
Current smoking, n (%)	Yes	206 (40.6)	507
	No	301 (59.4)	
Educational attainment, n (%)	No formal education	80 (15.8)	507
	Elementary school	106 (20.9)	
	Preparatory education	106 (20.9)	
	Secondary education	120 (23.7)	
	University/Higher education	95 (18.7)	
Self-reported economic status, n (%)	Below average	198 (39.1)	507
	Average	245 (48.3)	
	Above average	64 (12.6)	
Current residence (governorate), n (%)	Damascus & Damascus Suburb	131 (25.8)	507
	Aleppo	107 (21.1)	
	Homs	93 (18.3)	
	Lattakia	101 (19.9)	
	Hama	75 (14.8)	
Displacement status, n (%)	Original place	431 (85.0)	507
	Forcibly displaced	76 (15.0)	
Number of household members	**Median (IQR)**	**4.00 (IQR 3.00–5.00)**	507
Number of children	**Mean (SD)**	**3.24 ± 2.72**	507
Hemodialysis center (governorate), n (%)	Damascus & Damascus Suburb	128 (25.2)	507
	Aleppo	100 (19.7)	
	Hama	86 (17.0)	
	Homs	93 (18.3)	
	Lattakia	100 (19.7)	
Dialysis shift, n (%)	Morning	295 (58.2)	507
	Evening	212 (41.8)	
Companion during dialysis session, n (%)	Yes	404 (79.7)	507
	No	103 (20.3)	
Hemodialysis duration (months)	**Median (IQR)**	**36.00 (IQR 18.00–72.00)**	507
Duration of a single hemodialysis session (hours)	**Mean (SD)**	**3.53 ± 0.47**	507
Dialysis sessions per month	**Median (IQR)**	**8.00 (IQR 8.00–8.00)**	507
Time required to reach the dialysis center (minutes)	**Median (IQR)**	**30.00 (IQR 20.00–45.00)**	507
Dialysis as a financial burden, n (%)	Yes	271 (53.5)	507
	No	236 (46.5)	
Financial burden rating (1–10)	**Mean (SD)**	**6.51 ± 2.27**	271
Hypertension, n (%)	Yes	342 (67.5)	507
	No	165 (32.5)	
Diabetes mellitus, n (%)	Yes	147 (29.0)	507
	No	360 (71.0)	
Chronic pruritus, n (%)	Yes	221 (43.6)	507
	No	286 (56.4)	
Itching severity (1–10)	**Median (IQR)**	**5.00 (IQR 4.00–8.00)**	221
Hemoglobin (g/dL)	**Mean (SD)**	**9.10 ± 1.88**	466
History of blood transfusion, n (%)	Yes	329 (64.9)	507
	No	178 (35.1)	
History of infection related to hospital/dialysis, n (%)	Yes	151 (29.8)	507
	No	356 (70.2)	
Number of infections related to hospital/dialysis (count)	**Median (IQR)**	**1.00 (IQR 1.00–2.00)**	507
Regular physician follow-up, n (%)	Yes	381 (75.1)	507
	No	126 (24.9)	
Awareness of renal diet, n (%)	Yes	409 (80.7)	507
	No	98 (19.3)	
Adherence to renal diet, n (%)	Yes	258 (63.1)	409
	No	151 (36.9)	
Current erythropoietin (EPO) therapy, n (%)	Yes	380 (75.0)	507
	No	127 (25.0)	
CIASS Friends & Family	**Median (IQR)**	**4 (4–8)**	507
CIASS Work Colleagues	**Median (IQR)**	**12 (8–15)**	507
CIASS Healthcare Workers	**Median (IQR)**	**4 (4–7)**	507
CIASS Total	**Median (IQR)**	**24 (19–28)**	507
MSPSS Total	**Median (IQR)**	**67 (58–73)**	507
CIASS categories, n (%)	Anticipated stigma	201 (39.6)	507
	No stigma	306 (60.4)	
MSPSS categories, n (%)	Low support	12 (2.4)	507
	Medium support	156 (30.8)	
	High support	339 (66.9)	

Values are presented as n (%), mean ± SD, or median (IQR), as appropriate. Total *N* = 507 unless otherwise specified

### Clinical comorbidity and treatment-related characteristics

Hypertension was reported by 67.5% of participants and diabetes mellitus by 29.0%. Chronic pruritus was present in 43.6%, with a median severity of 5/10 (IQR 4–8). A history of blood transfusion due to renal failure was reported by 64.9% of patients, and 75.0% were receiving erythropoietin therapy (Table 1).

### Descriptive results for study scales

**Fig. 1:**
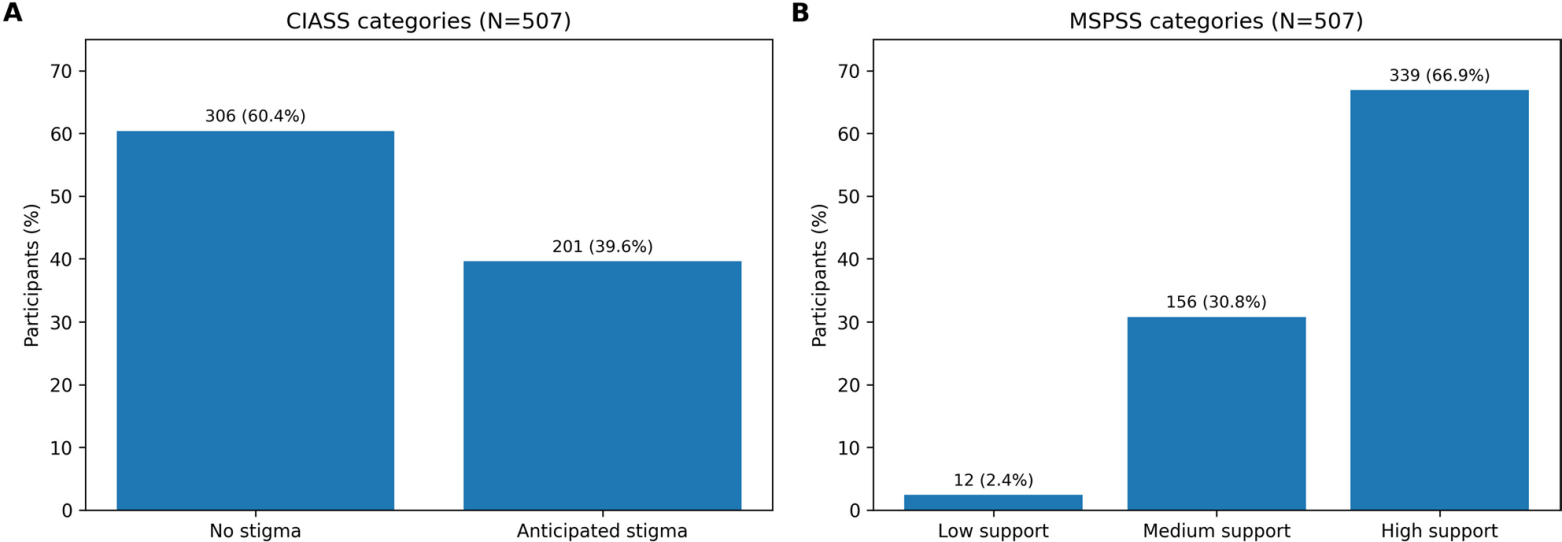
Distribution of anticipated stigma (CIASS) and perceived social support (MSPSS) categories (*N* = 507). Panel **A**: Proportion of participants classified as No stigma versus Anticipated stigma according to the Chronic Illness Anticipated Stigma Scale (CIASS). Panel **B**: Proportion of participants classified as Low, Medium, or High perceived social support on the Multidimensional Scale of Perceived Social Support (MSPSS). Bars represent percentages; labels above bars indicate n (%)

Median (IQR) scores were 24 (19–28) for the CIASS total and 67 (58–73) for the MSPSS total. Across CIASS domains, median (IQR) scores were 4 (4–8) for Friends and Family, 12 (8–15) for Work Colleagues, and 4 (4–7) for Healthcare Workers. Using the operational CIASS cut-off, anticipated stigma was identified in 39.6% of participants. Perceived social support was predominantly high, with 66.9% classified as having high support and only 2.4% classified as having low support (Table 1; Fig. 1).

### Bivariate associations

Perceived social support (MSPSS) was weakly lower with older age, longer hemodialysis duration, and more monthly sessions, and higher with larger household size (all *p* < 0.05). Overall anticipated stigma (CIASS total) was inversely correlated with MSPSS total (rₛ = −0.170, *p* < 0.001). Household size also showed domain-specific associations with CIASS, being inversely associated with Friends and Family stigma and positively associated with Work Colleagues stigma. Higher Work Colleagues stigma was associated with greater perceived dialysis financial burden (*p* = 0.007). In group comparisons, CIASS Healthcare Workers scores differed by displacement status (*p* = 0.003). Higher CIASS total scores were observed among participants reporting chronic pruritus, prior blood transfusion, and current EPO therapy (all *p* ≤ 0.001). MSPSS scores were higher among participants who had a companion during dialysis and among those receiving EPO (*p* ≤ 0.011). Both MSPSS and CIASS total scores varied significantly by current place of residence (MSPSS *p* = 0.001; CIASS total *p* < 0.001), with MSPSS highest in Damascus and Damascus Suburb and lowest in Latakia, while CIASS total was highest in Homs and lowest in Hama. MSPSS additionally differed across occupational categories (*p* = 0.033) (Table 2; Fig. 1).

**Table 2:** Bivariate Results

Outcome/Scale	Associated variable / grouping	Effect size	*p*-value	Direction statistic
MSPSS	Age	− 0.104	0.019	rₛ = − 0.104
MSPSS	Number of household members	0.148	0.001	rₛ = 0.148
MSPSS	Hemodialysis duration (months)	− 0.107	0.016	rₛ = − 0.107
MSPSS	Number of dialysis sessions per month	− 0.097	0.029	rₛ = − 0.097
CIASS: Friends & Family	Number of household members	− 0.149	0.001	rₛ = − 0.149
CIASS: Work Colleagues	Number of household members	0.124	0.005	rₛ = 0.124
CIASS: Work Colleagues	Rate of financial burden	0.165	0.007	rₛ = 0.165
CIASS Total	MSPSS Total	− 0.170	< 0.001	rₛ = − 0.170
CIASS Healthcare Workers	Original place of residence vs. forcibly displaced	0.134	0.003	The original place (*n* = 431), 261.41 Forcibly displaced (*n* = 76), 211.99
CIASS Total	Chronic itching (Yes vs. No)	0.145	0.001	Yes (*n* = 221), 258.76 No (*n* = 286), 250.33
CIASS Total	Blood transfusion due to renal failure (Yes vs. No)	0.258	< 0.001	Yes (*n* = 329), 281.73 No (*n* = 178), 202.74
CIASS Friends & Family	Blood transfusion due to renal failure (Yes vs. No)	0.100	0.025	Yes (*n* = 329), 263.81 No (*n* = 178), 235.78
MSPSS Total	Companion to the dialysis session (Yes vs. No)	0.144	0.001	Yes (*n* = 404), 264.86 No (*n* = 103), 212.13
CIASS Friends & Family	Level of Education	0.167	< 0.001	Z = 3.759
CIASS Work Colleagues	Level of Education	0.107	0.016	Z = -2.418
CIASS Healthcare Workers	Level of Education	0.188	< 0.001	Z = 4.226
CIASS Friends & Family	Economic Status	0.096	0.031	Z = 2.161
CIASS Work Colleagues	Economic Status	0.151	0.001	Z = -3.4
CIASS Healthcare Workers	Economic Status	0.135	0.002	Z = 3.041
MSPSS Total	Current place of residence	0.029	0.001	Damascus & Damascus Suburb (*n* = 131): 289.81 Aleppo (*n* = 107): 260.17 Homs (*n* = 93): 238.66 Lattakia (*n* = 101): 209.72 Hama (*n* = 75): 261.30
CIASS Total	Current place of residence	0.078	< 0.001	Damascus & Damascus Suburb (*n* = 131): 241.24; Aleppo (*n* = 107): 224.76; Homs (*n* = 93): 330.30; Lattakia (*n* = 101): 272.59 Hama (*n* = 75): 198.36
MSPSS Total	Current occupation	0.011	0.033	Office worker/office job (*n* = 110): 268.08; Retired (*n* = 182): 244.96; Never Worked (*n* = 117): 230.22; A job that requires physical effort (*n* = 98): 283.37
MSPSS Total	Currently receiving erythropoietin (EPO) therapy? (Yes / No)	0.113	0.011	Yes (*n* = 380): 263.55 No (*n* = 127): 225.42
CIASS Total	Currently receiving erythropoietin (EPO) therapy? (Yes / No)	0.174	< 0.001	Yes (*n* = 380): 268.70 No (*n* = 127): 210.02

### Multivariable regression analysis

A hierarchical binary logistic regression model (*N* = 507) was performed to identify independent correlates of anticipated stigma, defined as CIASS total score > 24. Sociodemographic variables alone did not significantly improve model fit (Block 1: Δχ² = 3.63, df = 12, *p* = 0.989; Nagelkerke R² = 0.010). Adding clinical and dialysis-related variables significantly improved model fit (Block 2: Δχ² = 23.39, df = 4, *p* < 0.001; Nagelkerke R² = 0.070). Adding hemodialysis hospital/governorate as a site-level covariate further improved the model (Block 3: Δχ² = 58.34, df = 4, *p* < 0.001; Nagelkerke R² = 0.210). Finally, adding MSPSS total score produced an additional significant improvement (Block 4: Δχ² = 16.57, df = 1, *p* < 0.001), yielding a final Nagelkerke R² of 0.246 with acceptable calibration (Hosmer–Lemeshow *p* = 0.546).

**Fig. 2:**
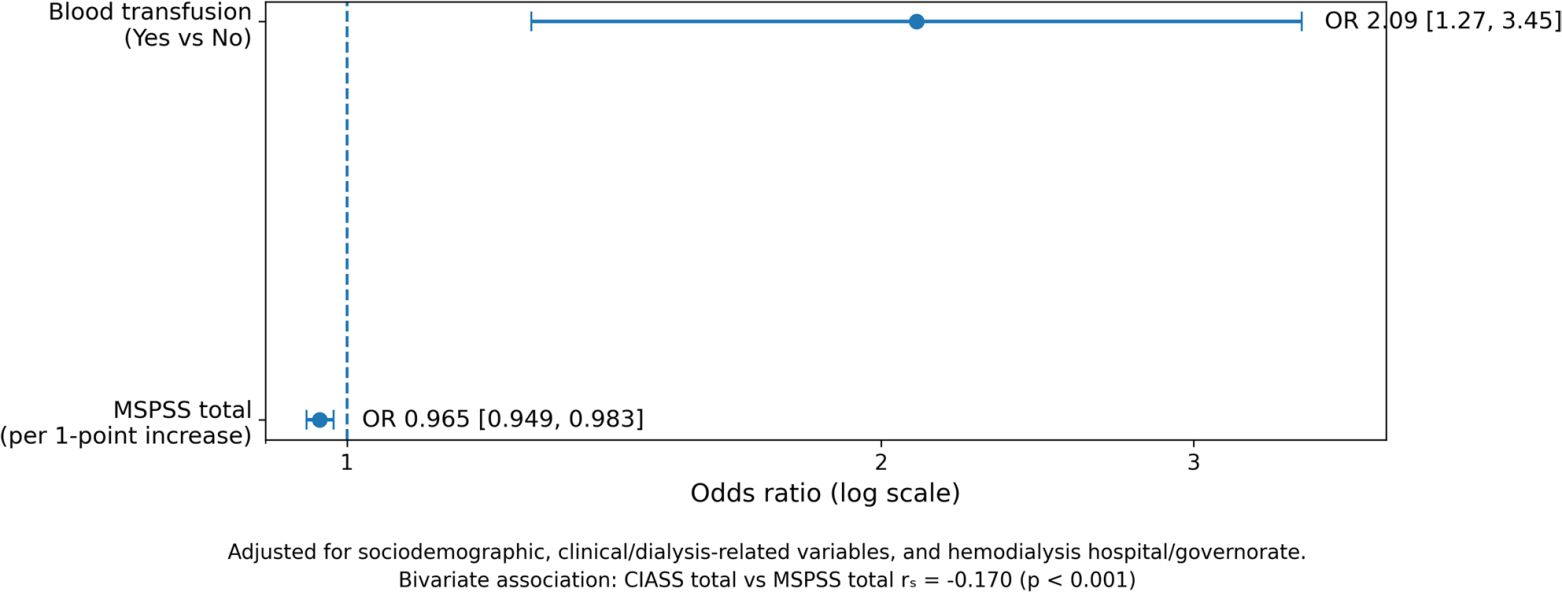
Adjusted correlates of anticipated stigma (CIASS category): final hierarchical logistic regression model (Block 4) (*N* = 507). Forest plot of adjusted odds ratios (ORs) and 95% confidence intervals (CIs) for variables retained in the revised final model predicting membership in the anticipated stigma category. Points indicate adjusted ORs and horizontal lines indicate 95% CIs on a logarithmic scale; the vertical dashed line indicates OR = 1.00 (no association). Higher odds of anticipated stigma were observed among participants with a history of blood transfusion due to renal failure (Yes vs. No; OR = 2.09; 95% CI 1.27–3.45; *p* = 0.004), whereas higher MSPSS total score was associated with lower odds of anticipated stigma (OR = 0.965 per 1-point increase; 95% CI 0.949–0.983; *p* < 0.001). The model was adjusted for sociodemographic variables, clinical/dialysis-related variables, and hemodialysis hospital/governorate

The final model was statistically significant overall (χ² = 101.92, df = 21, *p* < 0.001) and correctly classified 70.8% of participants, with a sensitivity of 59.2% and specificity of 78.4%. In the fully adjusted model, prior blood transfusion was independently associated with higher odds of anticipated stigma (OR = 2.09, 95% CI 1.27–3.45, *p* = 0.004). Higher MSPSS total score was independently associated with lower odds of anticipated stigma (OR = 0.965 per 1-point increase, 95% CI 0.949–0.983, *p* < 0.001). Hemodialysis hospital/governorate was also significantly associated with anticipated stigma in the final model (*p* < 0.001), while other sociodemographic and clinical covariates were not independently significant after adjustment (Tables 3 and 4; Fig. 2).

**Table 3:** Hierarchical binary logistic regression for anticipated stigma with hospital/governorate adjustment. Model fit by block

Block	Predictors entered	-2 Log Likelihood	Cox & Snell R²	Nagelkerke R²	Δχ² (Step)	df	*p*	Hosmer-Lemeshow p
1	Sociodemographic variables	677.320	0.007	0.010	3.628	12	.989	.118
2	Clinical/dialysis-related variables	653.933	0.052	0.070	23.387	4	<.001	.090
3	Hospital/governorate	595.591	0.155	0.210	58.342	4	<.001	.065
4	MSPSS Total	579.024	0.182	0.246	16.567	1	<.001	.546

Outcome: anticipated stigma, defined as CIASS total >24 (*N* = 507)

Final model: χ² = 101.924, df = 21, *p* < .001; sensitivity = 59.2%, specificity = 78.4%, overall accuracy = 70.8%

**Table 4:** Hierarchical binary logistic regression for anticipated stigma with hospital/governorate adjustment. Final model coefficients (Block 4)

Predictor	B	SE	Wald	df	*p*	OR	95% CI for OR
**Age**	-.007	.008	.934	1	.334	.993	0.978-1.009
**Number of household members**	-.031	.046	.441	1	.507	.969	0.886-1.061
**Level of Education**			0.621	4	.961		
No formal education vs university/higher education	-.269	.459	.344	1	.557	.764	0.311-1.879
Elementary school vs university/higher education	-.073	.397	.034	1	.855	.930	0.427-2.024
Preparatory education vs university/higher education	-.096	.379	.064	1	.800	.908	0.432-1.909
Secondary education vs university/higher education	-.189	.322	.344	1	.558	.828	0.440-1.556
**Economic Status**			0.795	2	.672		
Below average vs above average	.323	.391	.682	1	.409	1.381	0.642-2.972
Average vs above average	.278	.331	.705	1	.401	1.320	0.690-2.526
**Retired vs office job**	-.325	.307	1.122	1	.290	.723	0.396-1.319
**Never worked vs office job**	-.370	.352	1.103	1	.294	.691	0.346-1.377
**Physical-effort job vs office job**	-.452	.346	1.703	1	.192	.636	0.323-1.254
**Forcibly displaced vs original place**	-.152	.301	.255	1	.614	.859	0.476-1.550
**Dialysis financial burden**, Yes vs No	.404	.269	2.256	1	.133	1.498	0.884-2.538
**Chronic itching**, Yes vs No	.391	.227	2.969	1	.085	1.478	0.948-2.307
**EPO therapy**	.458	.327	1.967	1	.161	1.581	0.833-3.001
**Blood transfusion history**, Yes vs No	.739	.255	8.387	1	.004	2.094	1.270-3.451
**Hospital/governorate**			44.250	4	<.001		
Damascus & Damascus Suburb vs Lattakia	-.714	.426	2.810	1	.094	.490	0.212-1.129
Aleppo vs Lattakia	-.242	.436	.309	1	.579	.785	0.334-1.845
Hama vs Lattakia	-2.467	.520	22.523	1	<.001	.085	0.031-0.235
Homs vs Lattakia	.190	.433	.193	1	.660	1.209	0.518-2.825
**MSPSS Total**	-.035	.009	15.423	1	<.001	.965	0.949-0.983

Outcome: anticipated stigma, defined as CIASS total >24 *(N* = 507)

Reference categories were university/higher education, above-average economic status, office job, original place of residence, no financial burden, no chronic itching, no blood transfusion, and Lattakia for hospital/governorate

*OR* odds ratio, *CI* confidence interval, *MSPSS* Multidimensional Scale of Perceived Social Support

### Sensitivity analysis using CIASS total score as a continuous outcome

To address potential information loss from dichotomizing CIASS total scores, a sensitivity analysis was conducted using CIASS total as a continuous outcome with HC3 robust standard errors. The direction of the main associations was consistent with the logistic regression findings. Blood transfusion history was associated with higher CIASS total scores (B = 2.86, robust SE = 0.72, 95% CI 1.45 to 4.27, *p* < 0.001), whereas higher MSPSS total score was associated with lower CIASS total scores (B = − 0.083, robust SE = 0.027, 95% CI − 0.136 to − 0.029, *p* = 0.002). This supported the robustness of the main associations when CIASS was analyzed as a continuous score rather than as a dichotomized outcome.

## Discussion

### Principal findings

In this multicenter cross-sectional study of 507 maintenance hemodialysis patients in Syria, anticipated stigma was identified in 39.6% of participants based on the operational CIASS cut-off. Higher perceived social support was associated with lower odds of anticipated stigma in the fully adjusted model, whereas a history of blood transfusion was associated with higher odds. These two associations remained consistent in the sensitivity analysis that retained CIASS total score as a continuous outcome. The addition of hemodialysis hospital/governorate substantially improved model fit, indicating that anticipated stigma varied across dialysis settings and was not explained solely by individual-level sociodemographic or clinical characteristics. These findings reflect statistical associations only and do not imply causal relationships, given the cross-sectional design of the study.

### Conceptual clarification

This study specifically examined anticipated stigma, defined as expectations of future prejudice or discrimination related to chronic illness [12]. This construct is distinct from other stigma dimensions, including perceived, enacted, and internalized stigma [18]. Focusing on anticipated stigma captures expectations of future negative social evaluation that may influence disclosure, help-seeking, and social participation [12, 19]. Previous CIASS-based studies in chronic illness populations have shown that anticipated stigma can be measured across diverse settings, although estimates vary by population, instrument, and scoring approach [20, 21]. For example, Peltzer and Pengpid reported that 20.7% of chronic-disease patients endorsed anticipated stigma on at least one CIASS item [20], while Yeni found that anticipated stigma was associated with depression, reduced activities of daily living, and disruption of economic, social, and private life [21]. The 39.6% estimate in our cohort therefore adds evidence from a dialysis population in a conflict-affected setting, while direct comparison across studies remains limited by differences in operational definitions.

Although hemodialysis-specific research has rarely examined anticipated stigma explicitly, the broader dialysis literature consistently identifies stigma as a relevant psychosocial burden. Prior studies have shown that stigma-related constructs are associated with poorer quality of life and greater psychological distress in maintenance hemodialysis patients [6, 22]. However, these studies typically assess broader stigma dimensions rather than anticipatory expectations. The present study therefore extends this literature by focusing specifically on patients’ expectations of future negative social evaluation in a Syrian hemodialysis population.

### perceived Social support and anticipated stigma

Perceived social support was generally high in this cohort, consistent with evidence from dialysis populations in Middle Eastern settings where family-based caregiving structures are dominant [23, 24]. For example, studies in Saudi hemodialysis patients have reported high perceived social support, particularly from both family and friends [23]. In the present study, larger household size and the presence of a dialysis companion were associated with higher MSPSS scores in bivariate analyses, suggesting that practical caregiving and accompaniment may contribute to perceived support. However, high perceived support does not necessarily eliminate stigma-related expectations, as patients may simultaneously experience strong family support and fear of broader social judgment [25, 26].

Higher perceived social support was associated with lower anticipated stigma, but causality cannot be inferred due to the cross-sectional design. This inverse association is theoretically consistent with the expectation that, in hemodialysis populations, individuals with higher perceived social support may anticipate lower levels of negative social evaluation. These findings are consistent with evidence from hemodialysis and other chronic illness populations, where stigma- and support-related constructs have been linked to psychological distress, quality of life, and healthcare experiences [19, 27, 28]. Recent Syrian hemodialysis data also showed that social support is inversely associated with depression and stress, placing the MSPSS–CIASS association within a broader psychosocial context of distress among Syrian dialysis patients [9].

### Syrian context

The Syrian setting provides a critical contextual framework for interpreting the observed associations between anticipated stigma and perceived social support. In conflict-affected health systems such as Syria, structural disruptions may reshape patients’ psychosocial experiences by affecting continuity of care, patient–provider relationships, and perceived healthcare reliability, all of which are relevant to how individuals form expectations of social evaluation and perceive available support. The Health Stigma and Discrimination Framework conceptualizes stigma as a multilevel process operating across individual, interpersonal, and structural domains [29]. This framework is particularly relevant in Syria, where family-based support may coexist with forced displacement, economic strain, disrupted infrastructure, limited specialist access, and variable dialysis-unit conditions [8, 9, 30, 31].

From a stress-and-coping perspective, stigma and discrimination can act as identity-relevant stressors, while perceived social support is a relevant contextual factor [32, 33]. Importantly, these processes are not solely individual-level psychological mechanisms; they are embedded within broader relational and normative contexts that shape expectations of acceptance, evaluation, and social treatment. In particular, social psychological evidence shows that ingroup norms and belonging needs influence how individuals interpret others’ supportive or inclusive behaviors, indicating that such expectations are socially constructed rather than purely individual perceptions [34, 35]. Accordingly, these findings should be interpreted within this broader structural and relational context, which may shape how anticipated stigma and perceived social support are formed and expressed across settings.

### Clinical and contextual correlates

Blood transfusion history was independently associated with higher anticipated stigma in both the logistic regression model and the continuous CIASS sensitivity analysis. This finding is consistent with prior hemodialysis stigma research reporting associations between stigma and clinical burden, complications, and psychological distress [6]. This finding may reflect greater illness severity and healthcare exposure. In the Syrian context, where conflict-related renal care has been marked by shortages of supplies and medications, limited nephrology supervision, and disrupted service delivery [30, 31], transfusion may also indicate more complex disease management and greater reliance on healthcare systems. However, the present study did not measure the specific psychological or social meanings of transfusion, and therefore the underlying mechanism remains uncertain.

Other variables associated with anticipated stigma in bivariate analyses, including chronic pruritus, EPO therapy, financial burden, and selected sociodemographic factors, did not remain significant after multivariable adjustment. This attenuation suggests shared variance among clinical burden, socioeconomic vulnerability, and broader contextual and psychosocial factors, consistent with prior hemodialysis stigma research demonstrating that stigma is shaped by multiple interrelated determinants rather than isolated predictors [6]. Within this context, symptoms such as pruritus are likely better conceptualized as part of overall symptom burden rather than independent drivers of anticipated stigma.

### Displacement and site-level variation

Displacement showed a domain-specific association with healthcare-worker-related anticipated stigma in bivariate analysis but was not an independent predictor in adjusted models. This may reflect differences in care continuity and healthcare exposure patterns among displaced patients. In Northwest Syria, insecurity-related relocation disrupted dialysis continuity, led patients to use multiple dialysis sites, and coexisted with limited specialist access and variable service organization [8]. These disruptions may influence patient–provider interactions and continuity of care.

Dialysis hospital/governorate was a strong contributor to model fit, indicating meaningful variability across treatment settings. In conflict-affected health systems, such heterogeneity may reflect differences in resources, staffing, patient volume and continuity of care. Hemodialysis services are particularly vulnerable during war due to their dependence on uninterrupted access to equipment, supplies, trained staff, and safe transport [8, 30, 31, 36]. Evidence from other conflict settings, including Gaza and Sudan, shows that disruption of dialysis care is associated with interrupted or reduced treatment and worse patient well-being [36, 37]. These contextual factors likely explain the observed site-level variation in anticipated stigma, although specific institutional determinants were not measured in this study.

### Limitations

This study has several limitations. The cross-sectional design precludes causal inference and temporal interpretation between perceived social support, clinical factors, and anticipated stigma. Consecutive sampling from dialysis units may limit generalizability to patients not captured in this setting, and interviewer-administered questionnaires conducted by healthcare-affiliated personnel during dialysis sessions may have introduced social desirability and interviewer-related bias, which could have led to systematic underreporting of anticipated stigma and attenuation of observed associations, particularly for stigma-related responses.

Although the Arabic CIASS and MSPSS demonstrated good internal consistency following translation and pilot testing, full construct validation and measurement invariance were not assessed. The CIASS > 24 cut-off was used as an operational threshold rather than a validated clinical criterion; however, sensitivity analysis using the continuous score supported the robustness of the main findings. The work-colleagues domain may also be less applicable to non-employed participants, despite administration of the full scale to all respondents.

Importantly, key psychosocial confounders, including depression, anxiety, and broader psychological distress, were not measured or included in the multivariable models. These factors are likely to be associated with both perceived social support and anticipated stigma and may have influenced the observed associations. In addition, prior discriminatory experiences were not assessed and may represent relevant unmeasured determinants of stigma. The modest explanatory performance of the model further suggests that additional unmeasured factors likely contribute to anticipated stigma. Accordingly, the model is not intended for clinical screening or prediction and should be interpreted as exploratory rather than diagnostic.

Finally, although hospital/governorate was included in the model, potential clustering effects were not formally accounted for using multilevel modeling, which may have resulted in residual center-level confounding.

### Implications for future research and care

In clinical practice, brief psychosocial screening and patient-centered communication, with attention to family or companion involvement, may help identify patients at higher risk of anticipated stigma, particularly those with greater illness burden such as transfusion history. Future longitudinal studies are needed to clarify temporal relationships between social support and anticipated stigma. Further research should also incorporate key unmeasured factors, including depression, anxiety, prior discrimination experiences, internalized stigma, and detailed center-level characteristics, to better elucidate the pathways linking clinical burden, social support, and stigma in hemodialysis populations.

## Conclusion

This study documents a substantial burden of anticipated stigma among patients receiving maintenance hemodialysis in Syria and identifies an inverse association with perceived social support. Blood transfusion history was also associated with higher anticipated stigma and may reflect greater illness burden, although underlying mechanisms remain uncertain. Given the cross-sectional design and modest model performance, these findings should be interpreted as associative rather than causal. They nevertheless highlight the importance of psychosocial and contextual factors in hemodialysis care in conflict-affected settings. Further longitudinal and context-sensitive research is needed to clarify underlying pathways and inform appropriate stigma-reduction strategies. 

## Data Availability

The datasets generated and/or analysed during the current study are not publicly available due to privacy and ethical restrictions but are available from the corresponding author on reasonable request.
